# Effects of Argon-Based and Nitrogen-Based Modified Atmosphere Packaging Technology on the Quality of Pomegranate (*Punica granatum* L. cv. Wonderful) Arils

**DOI:** 10.3390/foods10020370

**Published:** 2021-02-09

**Authors:** Ilenia Tinebra, Dario Scuderi, Giuseppe Sortino, Paolo Inglese, Vittorio Farina

**Affiliations:** Department of Agricultural, Food and Forest Sciences (SAAF), Università degli Studi di Palermo, Viale delle Scienze, 90128 Palermo, Italy; ilenia.tinebra@unipa.it (I.T.); dario.scuderi@unipa.it (D.S.); giuseppe.sortino@unipa.it (G.S.); paolo.inglese@unipa.it (P.I.)

**Keywords:** fruit quality, sensory analysis, postharvest technologies, cold storage, modified atmosphere, noble gases

## Abstract

Ready-to-eat pomegranate arils are considered a “functional food” for their health benefits and have desirable sensory characteristics, which have caused an increasing interest by the consumers for this product. The preparation process of ready-to-eat fruit products can cause severe injuries and worsen their quality and shelf life significantly. Modified atmosphere packaging (MAP) has been used broadly in the last years to maintain the quality of processed fruits and showed optimal results, in spite of the possible problems caused by the depletion of O_2_ and corresponding accumulation of CO_2_ in the package. This study was conducted to evaluate the effects of different MAP treatments, based on nitrogen or alternatively on a noble gas, argon, in combination with refrigerated storage (0, 4, 8, 12, and 16 days at 4 ± 1 °C and 90 ± 5% RH) on the qualitative parameters of pomegranate arils with the aim to prolong their post-harvest life maintaining the original quality. The argon-based MAP treatment (MAPAr) was the one that provided the best results, assuring a limited loss of weight and juice content. The use of noble gas allowed to maintain a high sugar/acid ratio until 16 days from packaging. Sensory analysis on all MAP treated arils and, on the juice, obtained from them were carried out, and judges showed a preference for MAPAr treated arils and juice until day 12 from packaging.

## 1. Introduction

Commercial farming of pomegranates increased globally in the last decade, due to the renowned health benefits and nutritional characteristics of the fruit, especially in the Mediterranean basin, thanks to improvements in the agronomic practices under which the crop is cultivated [1,2] and to the introduction of new world-class cultivars such as Wonderful [3]. Consumption of the fresh pomegranate fruit, though, still encounters some resistance in the consumers due to the large amount of arils every fruit bears and the difficulty of extracting them from the fruit, due to the presence of phenolic metabolites, which can stain the hands during preparation of seeds [4]. One viable solution for the placing of the product on the market is the preparation of various packaged items containing ready-to-eat pomegranate arils. Such products also allow the producer to obtain a good price, even for fruits that show external appearance flaws like sunburnt husks, splits, and cracks, and offer to the consumer a product with high nutritional value, good taste, and great eating convenience [5,6,7]. Therefore, ready-to-eat arils also offer relevant social and economic opportunities for growing regions, by adding value to the final products and creating employment for the high quantity of required work, and might let the fruit be processed even after long storage periods [8].

Many efforts are being made to manage maintaining the nutritional and microbial quality of pomegranate arils, because even when minimally processed, they tend to easily deteriorate in texture, color, and overall quality and present an unsatisfying shelf life [9].

Kader et al. [10] describe pomegranate as a non-climacteric fruit due to its low respiration and ethylene production rates after harvest. Nonetheless, its shelf life is relatively short due to a rapid quality loss over time, and browning is the major physiological disorder that affects the sensory properties of ready-to-eat arils [11].

Various treatments have been tested to preserve quality and extend the shelf life of pomegranate whole fruit and arils, such as intermittent warming, curing, film wrapping, waxing, application of polyamines, honey treatments, controlled atmosphere, and modified atmosphere packaging (MAP), and they produced mixed results [2,11,12,13,14,15,16]. MAP relies on the dynamic process of alteration of gaseous composition inside a package determined by permeability of packaging film and produce respiration. Combined with low temperature storage, it has been successfully used to prolong the shelf life of fresh fruit and vegetables. The use of MAP reduces the respiration rate and activity of insects or microorganisms and provides control of fruit and vegetable ripening, or browning in cut produce, and ultimately extends the shelf life [17,18].

With regards to packaging systems for MAP, various films have been tested over the years [19]. For example, Palma et al. [8] used polypropylene film bags, and managed maintaining good quality of arils after 10 days of shelf life; Sepúlveda et al. [20] observed that minimally processed pomegranate arils cv. “Wonderful” were storable for 14 days in semipermeable films; the same material was used by Gil et al. [9], who reached seven days of shelf life, and Banda et al. [21] obtained a shelf life of 12 days for arils of Wonderful cv. arils using polypropylene clam shell trays. Artés et al. [22] reported that MAP strongly contained water loss and chilling injuries without incidence of decay, under a gas composition of 8% O_2_ and 10% CO_2_ at 5 °C. Caleb et al. [23] pointed out that after the seventh day of storage, fungal growth and off-odor prejudiced consumer appreciation of MAP-treated pomegranate arils. Moreover, irradiation with UV-C rays before sealing of the MAP packages could not prolong the arils’ post-harvest life beyond 10 days when the legal microbial charge threshold was also reached [24]. Nevertheless, postharvest loss of pomegranate fruits can often exceed an incidence of 30% of the produce for some cultivars [25]. Therefore, more alternatives are needed to obtain a reduced microbial charge of MAP-stored pomegranate arils and delay quality loss.

Unconventional MAP with argon could represent a valid solution to maintain post-harvest pomegranate arils quality, keeping microbial charge below security levels. Many studies have investigated the potential benefits of argon (Ar) in MAP applications in recent years [26,27,28]. Contrasting results, though, were obtained when investigating the effect of Ar on inhibition and control of the growth of some microorganisms, on the activity of food quality-related enzymes, and on degradative chemical reactions in perishable food products [26,27,29,30,31,32,33]. For example, kiwifruit slices packed in modified atmospheres with 90% of Ar compared to those packed in air and in N_2_ at the beginning of storage showed a less intense CO_2_ production, but had a worse appearance and overall consumer acceptability [34]. The stronger ability of Ar to retard fruit physiology, compared with N_2_, could be a consequence of the higher capacity of the noble gas to dissolve in the aqueous layer of the cut fruit and through pulp cells consecutively. It is possible, then, that Ar is more effective than N_2_ in inactivating some chemically active sites on the enzymes and reducing the level of dissolved oxygen, which oxidative enzymes need to catalyze metabolic reactions [27,30,31,33,35]. As for the economic viability of the use of Ar for the MAP treatment of pomegranate arils, it has to be considered that the cost of the gas (about 2.40 € per liter) can be justified by the high market price of ready-to-eat arils, which varies from 12 to 20 € per kilogram [36].

The aim of this research was to evaluate the effectiveness of Argon as component of MAP gaseous mixture combined with cold storage (0, 4, 8, 12, and 16 days at 4 ± 1 °C and 90 ± 5% RH) in maintaining microbiological, physico-chemical, and sensory quality of pomegranate arils (cv. Wonderful) packaged in low density polyethylene film bags.

## 2. Materials and Methods

### 2.1. Vegetal Materials

**Figure 1:**
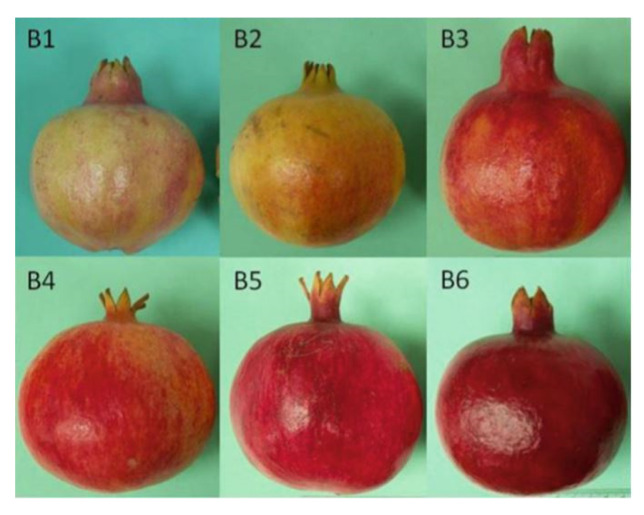
Ripening stages of pomegranate fruit according to Holland and Bar-Ya’akov’ [37].

Pomegranate fruit (*Punica granatum* L.) cv. Wonderful was harvested at commercial ripening stage when they were found to belong to category B2, assessed through epicarp color analysis, in the classification of Holland and Bar-Ya’akov’ [37] (Figure 1), from a farm located in Naro (37°17′14.0″ N 13°47′30.1″ E), province of Agrigento, Sicily. This cultivar was chosen, as the most widely cultivated in Sicily and one of the most consumed in Europe, for the characteristics of its arils, which fit minimally processed transformation [38,39,40,41]. After harvest, the fruit was transferred to the post-harvest laboratory and processed within 24 h. Fruit that did not present visible physical defects were washed in sterilized water, and arils were extracted manually with a curved knife and washed in chlorinated water and 3% citric acid for 2 min.

Physical characteristics of whole fruit were measured on a representative sample of 10 fruit at the beginning of the study for the following parameters: fruit weight (g); fruit length—longitudinal diameter (cm); fruit width—transverse diameter (cm); fruit volume (cm^3^); *Shape index*, obtained as (Equation (1)):

(1) $$Shape\;index=\;\frac{TD}{LD}$$

where *TD* refers to transverse diameter and *LD* is longitudinal diameter; skin color (CIE L*a*b*: lightness (L*), redness/greenness (a*), and yellowness/blueness (b*)); skin weight (g); skin thickness (cm); *Skin index*, obtained as (Equation (2)):

(2) $$Skin\;index=\;\frac{SW}{FW}$$

where *SW* is the skin weight and *FW* is the fruit weight. Data are presented in (Table 1). 

Later on, fruit were opened with a curved knife and arils were manually extracted and went through the evaluations of number of arils; weight of arils per fruit (g); weight of 20 arils (g); aril height—longitudinal diameter (cm); aril width—transverse diameter (cm); aril yield, obtained as fruit weight—skin weight (g); weight of 20 seeds (g). Data are presented in (Table 2).

Moreover, characteristics of the juice extracted from the arils using a centrifugal juicer (Centrika Metal, Mod. 0173, Ariete, Firenze, Italy) were measured: juice content (mL/100 g); total soluble solids content (°Brix); titratable acidity (g L^−1^ citric acid); pH. Data are presented in (Table 3).

Weight of arils was measured with a precision electronic scale accurate to two decimal places (Gibertini EU-C 2002 RS, Novate Milanese, Italy), while the diameter and length of the fruit were measured with a digital caliper (Turoni TR53307, Forlì, Italy). Volume of the whole fruit was determined by immersing it in a 2000 mL graduated cylinder filled with water. Characteristics of whole fruit agree with the requirements for export of fresh pomegranate to Europe. Fruits were found belonging in UNECE category 1/A [39]. All analyses results are presented as mean ± standard deviation.

### 2.2. Experimental Design

Fruit were manually selected, removing those which presented blemishes, including overripe or too small fruit. After that, 36 fruits were selected randomly, and each whole fruit was washed with distilled water (5 °C) and sanitized in 200 μLL^−1^ Ox-Virin (solution of hydrogen peroxide and peroxyacetic acid; 0.5% *w*/*v*) for 2 min.

Fruits were later opened with a curved knife, and arils were washed in chlorinated water and 3% citric acid for 2 min, extracted, and collected in sterile containers to allow drying and manually remove the damaged ones. Arils from all fruits were put together and portions of 200 g (about 630 arils) from the homogeneous arils mix were weighed and sealed into low density polyethylene film bags (LDPE, Orved, S.p.A., Musile di Piave, Venezia, Italy).

Characteristics of the LDPE 90*μ*-80 mm-500 cm^3^ films were the following: permeation to O_2_ (cm^3^ m^−2^ day^−1^): 4050; permeation to CO_2_ (cm^3^ m^−2^ day^−^^1^): 14,000; OTR: 7500 cm^3^ m^−2^ day^−1^; WVTR: 6.5 g m^−2^ day^−1^. Modified atmosphere was obtained inside the sealed bags, using a digitally controlled packaging machine (VM 16 Orved S.p.A, Musile di Piave, Venezia, Italy).

Samples were divided as follows: 200 g of arils per bag × 3 bags × 3 treatments × 5 storage times. Therefore, a total of 45 bags was used.

To understand the effect of modified atmosphere packaging (MAP), three treatments were applied: one control passive-MAP treatment (CTR), one nitrogen-based treatment (MAPN_2_) and one argon-based treatment (MAPAr). Five storage times were tested: 0 (T_0_), 4 (T_4_), 8 (T_8_), 12 (T_12_), and 16 (T_16_) days of cold storage at 4 ± 1 °C and 90 ± 5% RH.

The three treatments were based on the following gaseous mixtures:

- CTR: 20.9% O_2_ + 0.04% CO_2_ (passive-MAP);
- MAPN_2_: 10% O_2_ + 5% CO_2_ + 85% N_2_;
- MAPAr: 10% O_2_ + 5% CO_2_ + 85% Ar.

The relationship between the quantity of product and the gas mixture injected was 1:2 (*V*/*V*). Fruit, headspace of the package, and the surrounding environment were initially at the same temperature.

To investigate the microbial and physicochemical properties of pomegranate fruit samples, analyses were conducted on fresh arils prior to storage (T_0_). Sampling for further microbiological, physico-chemical, and sensory analyses was made at each tested storage time. Each package was weighed again before conducting the analysis of arils at each storage time.

### 2.3. Physico-Chemical Analysis

Net weight of arils contained in the bags was measured using a precision electronic scale accurate to two decimal places (Gibertini EU-C 2002 RS, Novate Milanese, Italy), with an accuracy of ±0.01 g. Using the initial weight of sample recorded prior to storage, weight loss (WL) was calculated using (Equation (2)) as formulated by Belay et al. [42]:

(3) $$WL=\frac{W_{0}-W_{f}}{W_{0}}\times 100$$

where *WL* is the weight loss (%), *W*_0_ is the initial weight (g), and *W_f_* is the final weight (g) prior to package analysis.

A total of 50 g of arils from each pack were squeezed using a centrifugal juicer (Centrika Metal, Mod. 0173, Ariete, Firenze, Italy) to extract the juice. Initially, the juice content of 50 g of arils per packet was evaluated, and then chemical characteristics were analyzed. Pomegranate juice pH and titratable acidity (TA) were measured using a pH meter-titrator (Titromatic 1S, Crison, Barcelona, Spain). Titratable acidity (TA) was expressed as grams of citric acid per liters of crude pomegranate juice (g L^−1^ citric acid) and was determined by titration to an end point of pH 8.2 using 5 mL of juice diluted with 10 mL distilled water. Total soluble solids content (TSSC) were measured by a digital refractometer (Atago, Tokyo, Japan) and expressed as °Brix, and finally the total soluble solids content/titratable acidity ratio was calculated. All analyses were presented as mean ± standard deviation (SD) of three replicates.

### 2.4. Colorimetric Analysis

Color of arils was determined on basis of CIE L*a*b* color system measured using a digital colorimeter (CR-400 Chroma Meter, Minolta, Japan). Calibration of the color meter was performed against a white tile background (Illuminants C: Y ¼ 89.53, x ¼ 0.3247, y ¼ 0.3198) prior to each measurement.

Twenty grams of arils were weighed into a petri dish, and the color of the arils was measured on five different points of the dish [43]. Color parameters (L*, a*, and b*) were measured, and means of all measurements were determined for each package. Color difference (ΔE) between the colors at each evaluation time for the arils of each treatment and the color of the fresh arils measured at T_0_, following the (Equation (4)):

(4) $$\Delta E=\sqrt{{\left(L^{*}-L_{0}^{*}\right)}^{2}+{\left(a^{*}-a_{0}^{*}\right)}^{2}+{\left(b^{*}-b_{0}^{*}\right)}^{2}}$$

where L*, a*, and b* and $L_{0}^{*}$, $a_{0}^{*}$, and $b_{0}^{*}$ represent the current and initial values of the color of the arils. Chroma (C*) values, which indicate the quantitative attribute of color intensity, and hue angle (*h*°), which is considered as the qualitative attribute of color of samples, were calculated using (Equations (5) and (6)), respectively:

(5) $$C^{*}=\;\sqrt{\left(a^{*2}+b^{*2}\right)}\;$$

(6) $$h{}^{\circ}=\arctan\left(\frac{b^{*}}{a^{*}}\right)$$

Analyses were presented as mean ± standard deviation (SD) of three replicates.

### 2.5. Headspace Gas Composition

Before opening the packages at each storage time, the gas composition (oxygen and carbon dioxide) inside the bags was determined using a gas analyzer (PBI Dansensor, Ringsted, Denmark). Gas analysis was performed by inserting a needle attached to the gas analyzer through an adhesive seal fixed on the lidding material. The measurements were taken at two different sides of each package. The results were represented as O_2_ and CO_2_ percentages. All analyses were presented as mean ± standard deviation (SD) of two replicates.

### 2.6. Microbiological Analysis

Microbiological analysis was conducted following the work of Passafiume et al. [44]. Different microbial groups were investigated as follows: total mesophilic microorganisms (TMM) on plate count agar (PCA), total psychrotrophic microorganisms (TPM), and yeasts and molds on yeast extract peptone dextrose (YPD) agar supplemented with 0.1-g/L chloramphenicol to avoid bacterial growth. All analyses were presented as mean ± standard deviation (SD) of three replicates.

### 2.7. Sensory Evaluation

Sensory evaluation was performed by a team of 30 judges (sixteen men and fourteen women aged between 20 and 60 years) with a good background and knowledge of the details of this kind of food evaluation [45], following the guidelines of UNI 10957:2003 legislation [46]. During the preliminary meetings, 20 qualitative descriptors were chosen for the definition of the sensory profile, nine descriptors for arils and 11 descriptors for juice, generated based on citation frequency (>60%) and listed below:

For arils: color, pomegranate odor, herbaceous odor, acid, sweet, bitter, astringent, pomegranate flavor, herbaceous flavor.

For juice: color, density, pomegranate odor, herbaceous odor, acid, sweet, bitter, astringent, pomegranate flavor, herbaceous flavor, overall evaluation.

The evaluation was carried out from 10.00 a.m. to 12.00 p.m. in a room under white lights. Each panelist received in random order a sample of 10 arils and 20 mL of juice, and water was provided for rinsing the mouth between each sample.

The judges evaluated the intensity of each descriptor by assigning a score between 1 and 9, where each score represented a different level of intensity of the quality descriptors. The panelists assigned scores to the descriptors according to the nine-point intensity scale: 1—no sensation, 2—barely recognizable, 3—very weak, 4—weak, 5—light, 6—moderate, 7—intense, 8—very intense, and 9—extremely intense [45,47].

### 2.8. Statistical Analysis

Two-Way ANOVA was performed to evaluate the effect of the MAP storage period and of the independent treatments on the quality parameters using the univariate general linear model procedure of Systat software Inc. (Chicago, IL, USA). Significant differences (*p* ≤ 0.05) between treatments and during time of storage were evaluated by Tukey’s multiple range test (Tukey HSD test). Pearson’s correlation was performed using software Systat software.

## 3. Results and Discussion

### 3.1. Physico-Chemical Analysis

**Figure 2:**
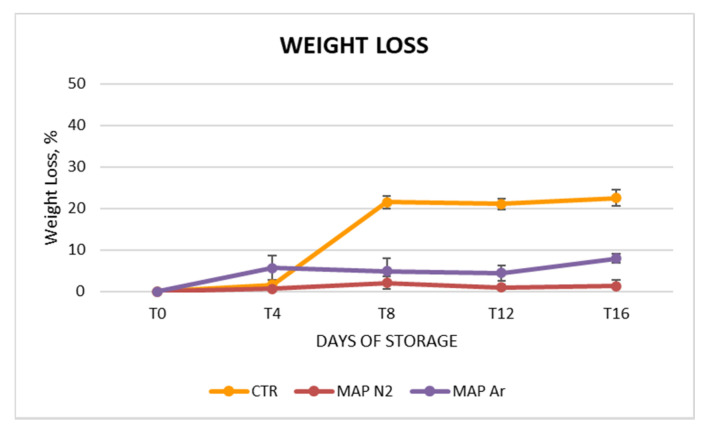
Trend of weight loss (%) of arils during cold storage and treated with MAP (CTR: 20.9% O_2_ + 0.04% CO_2_ (passive-MAP); MAPN_2_: 10% O_2_ + 5% CO_2_ + 85% N_2_; MAPAr: 10% O_2_ + 5% CO_2_ + 85% Ar). Values were recorded at 0 (T_0_), 4 (T_4_), 8 (T_8_), 12 (T_12_), and 16 (T_16_) days at 4 ± 1 °C and 90 ± 5% RH. Values represented as mean ± SD. of each treatment for each date.

The treatment that showed the greatest net weight loss was CTR (Figure 2); the weight of untreated arils dropped below 80% of its initial weight already after eight days of storage. In contrast, weight loss for MAPAr treatment reached values of about 5% after day 4 and until day 12 of storage. On the other hand, the treatment that best limited weight losses was MAPN_2_, where they amounted to less than 2% until T_16_ (final weight = 98.7% of initial value). Similar results were reported by Caleb et al. [23] who reported a weight loss of 0.53% of arils (cv. Acco) packed in MAP after 14 days of storage at 5 °C. The weight retention for MAPN_2_ and MAPAr arils could be due to the higher retention of moisture than CTR, which allowed maintain the arils structure intact [48].

**Figure 3:**
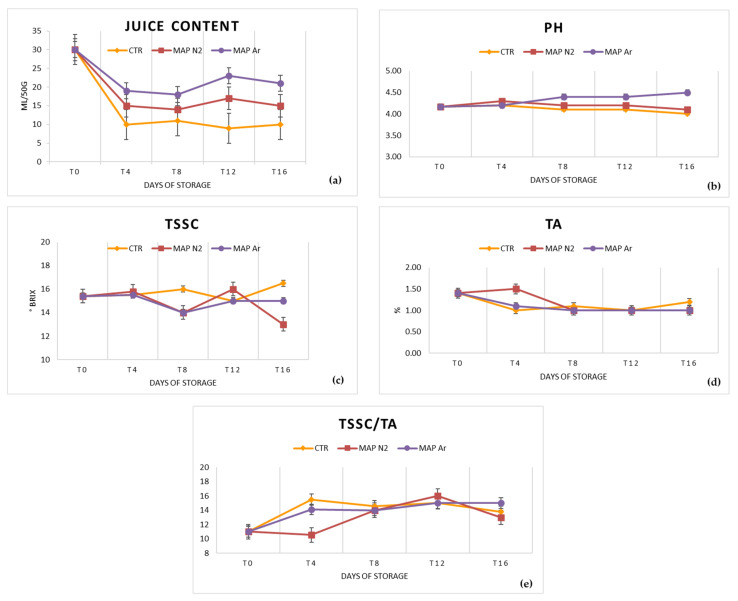
Results of the analyses of (**a**) juice content (mL/50 g); (**b**) pH; (**c**) TSSC (total soluble solids content, °Brix); (**d**) TA (titratable acidity, g L^−1^ citric acid); (**e**) TSSC/TA ratio treated with MAP (CTR: 20.9% O_2_ + 0.04% CO_2_ (passive-MAP); MAPN_2_: 10% O_2_ + 5% CO_2_ + 85% N_2_; MAPAr: 10% O_2_ + 5% CO_2_ + 85% Ar). Values were recorded at 0 (T_0_), 4 (T_4_), 8 (T_8_), 12 (T_12_), and 16 (T_16_) days at 4 ± 1 °C and 90 ± 5% RH. Values represented as mean ± SD.

Although all samples showed a decrease in juice content of the arils (Figure 3a), MAPAr was the treatment that best maintained the juiciness, with values decreasing from 30 to 21 mL juice per 50 g arils after 16 days of storage. The CTR arils had a value of juice content decreasing from 30 to only 10 mL of juice per 50 g of arils during storage, while the MAPN_2_-treated arils had an intermediate result, with juiciness decreasing from 30 to 15 mL per 50 g of arils after 16 days of storage (Figure 3a).

Generally, the pH values of arils juice did not vary significantly in any of the treatments throughout the storage period (Figure 3b) only the MAPAr samples reached a pH of 4.5 at T_16_, slightly higher than the value at T_0_. The treatments MAPN_2_ and CTR showed a decrease starting from T_4_ that was not statistically significant (*p* ≥ 0.05). The initial and subsequent pH values of the arils were slightly higher than those reported by several other authors [21,41,42], who reported pH values not exceeding 4. Fluctuations in arils pH can be linked to differences in CO_2_ accumulated in the packages during storage. A similar trend was observed by Ayhan and Eştürk on arils subjected to passive MAP cold storage [49]. Artés et al. [43] reported that no MAP treatment led to a decrease in pH values of pomegranate arils after shelf life.

All treatments showed a fluctuating trend of TSSC and, as Figure 3c shows, a decrease occurs in both MAP treatments between T_4_ and T_8_, while increasing between T_8_ and T_12_. Later, MAPN_2_ is the only treatment that shows a remarkable decrease in its TSSC after 12 days of storage, reaching, at T_16_, a value below 14 °Brix. Instead, CTR is the only treatment showing a relevant increase of its TSSC from the initial value of 15.4 °Brix, while MAPAr treated samples maintained values alike to the initial one throughout the storage period, with a final value of 15 °Brix. The decrease in TSSC in MAPN_2_ could be attributed to an increase in metabolic activities of pomegranate arils during storage, such as the conversion of soluble sugars [50]. Additionally, Bhatia et al. [50] observed a similar trend of decreasing TSSC of pomegranate arils packed in LDPE MAP films. The increase in TSSC in CTR, on the other hand, could be due to the combined effect of water loss due to high dehydration [9] and the marked decrease in O_2_ that was observed in this treatment [51]. The range of TSSC values we observed was in line with that reported by several other authors [21,52].

As Figure 3d shows, MAPAr and CTR showed a similar trend during all storage periods with regards to TA, which decreases from day 0 to day 4 and later remains practically unchanged. Values of TA for all treatments during the storage period were between 1% and 1.5%, a range similar to that reported by most other authors for cv. Wonderful arils [53,54]. Only in MAPN_2_ could it be observed that TA decreased significantly at day 8 (T_8_) and remained almost constant for the remaining storage period. The decrease in acidity during storage was observed also by Artés et al. and Caleb et al. [23,43] and could be related to the metabolic activities of the fruit, and, as reported by Fagundes et al. [55], to the use of organic acids as a substrate in the respiration or transformation of sugars.

This resulted in a TSSC/TA ratio that, at the end of the storage period, was highest in MAPAr-treated arils with a value of 15, intermediate in untreated CTR arils (13.75), and lowest in MAPN_2_ treatment (13), values higher than those found on the same cv. by Banda et al. [21] (Figure 3e).

### 3.2. Colorimetric Analysis

Table 4 shows that there is significant effect of MAP (*p* > 0.05) especially on the a∗ parameter (redness). Both CTR and MAPN_2_ suffered, during the entire storage period, an important fluctuation of this value, starting from T_4_. MAPAr-treated fruits, on the other hand, kept the value of a* constant during the entire storage period, thus maintaining a stable aril color appearance.

The effects of MAP and storage time were insignificant on the b* parameter values of arils (*p* > 0.05), although small fluctuations could be observed. There were significant effects of MAP application on L* (brightness) values (*p* ≤ 0.05). Both CTR and MAPN_2_ go through a decrease of L* value during the 16 days of storage. MAPAr, instead, maintains brightness values similar to those of the fresh fruit up to day 12 of storage. A similar behavior was observed by Artés and Tomas-Barberan [43], who showed that storage of arils with MAP leads to pigment stability.

Values of ΔE show that the treatment that caused the smallest variation from the initial color of the arils was MAPAr, where ΔE stays below 3 until day 12 of storage (T_12_). CTR was the treatment that caused the biggest deviation from the original color of the arils, while MAPN_2_ maintained intermediate values. Values of Chroma (C*) of all treatments stayed around a median value of 20 throughout the storage, whereas MAPN_2_ had much more fluctuating values compared to the other treatments, probably because of the insurgence of browning symptoms. A slight decrease in C* was observed in MAPN_2_-packed arils from T_12_, while MAPAr-packed arils maintained stable C* values throughout the storage period. This is different from what was obtained by other authors, who reported Chroma values of pomegranate arils in MAP storage decreasing during storage [53,56]. The hue angle (*h*°) decreased slightly in all arils bags starting from T_4_, except for MAPAr, where the values remain constant until T_12_ [52]. De Reuck et al. [57] noted that C* and *h*° values decreased during 21-days long MAP cold storage at 2 °C of fresh lychees. These behaviors could be due to the excess of CO_2_ in the packages, as reported by Hussein et al. [53].

### 3.3. Headspace Gas Composition

**Figure 4:**
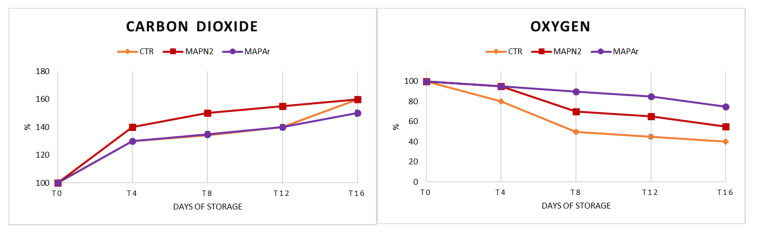
Effect of MAP on the headspace gas concentration (%) during storage (CTR: 20.9% O_2_ + 0.04% CO_2_ (passive-MAP); MAPN_2_: 10% O_2_ + 5% CO_2_ + 85% N_2_; MAPAr: 10% O_2_ + 5% CO_2_ + 85% Ar). Values were recorded at 0 (T_0_), 4 (T4), 8 (T_8_), 12 (T_12_), and 16 (T_16_) days at 4 ± 1 °C and 90 ± 5% RH. Data correspond to the means ± SD.

During cold storage, the gas composition changed significantly (*p* < 0.05) from the initial value in the bags of all treatments, as shown in Figure 4. A decrease in the CO_2_ content and a corresponding increase in the O_2_ concentration was observed in all treatments, as observed by Baley et al. at the storage temperature of 5 °C [58]. Results show that CO_2_ increased to a greater extent in CTR and MAPN_2_ bags, in particular after day 4. The bags containing the MAPAr gaseous mixture show a less pronounced increase of CO_2_ values, especially after T_4_, when CO_2_ production rates slow down. Caleb et al. [59] reported that the increase in CO_2_ percentages was linked to the production of off-odor and off-flavor at the opening of the packages. Therefore, the use of Ar could limit the occurrence of this phenomenon, as it was confirmed by the sensory analyses.

In CTR packages, O_2_ concentration shrinks consistently compared to MAPN_2_ and MAPAr samples after T_4_. The level of O_2_ reached by CTR is detrimental, because it promotes anaerobic fermentative reactions leading to unpleasant odors and off-flavors [44]. O_2_ concentration decreases in both MAP treatments with a smaller rate, suggesting a slower respiration rate from the arils inside the bags. MAPAr, in the end, was the treatment that allowed maintaining the highest percentage of O_2_ (80%) within the sealed packages.

### 3.4. Microbiological Analysis

The presence of total mesophilic (TMM) and psychrotrophic (TPM) bacteria on arils increased significantly after T_12_ (Table 5). The three treatments had different influences on the growth of all microorganisms analyzed (*p* < 0.05).

The lowest counts of aerobic mesophilic bacteria were observed in MAPAr and the highest counts of packed arils in CTR. Similarly, the lowest counts of yeasts and molds were observed in MAPAr, although the latter only appeared at T_16_. Caleb et al. [23] observed similar results on passive MAP of cold stored arils, reporting that aerobic mesophilic bacteria were less present than yeast and mold.

The higher yeast and mold counts observed in MAPN_2_ and CTR (Table 6) packed arils could be attributed to the higher level of CO_2_ recorded during storage. In fact, some studies suggest that the presence of yeast and mold counts in packaged products may be promoted by the increase or accumulation of CO_2_ inside the packages [24].

Our results are in agreement with several studies that showed that MAP with Argon effectively reduced the microbial load, extending the shelf life by up to 15 days [60,61]. In addition, the maintenance of high O_2_ levels in MAPAr can be inferred to have been effective in inhibiting anaerobic reactions and anaerobic microbial growth.

### 3.5. Sensory Analysis

Fresh raw arils were well accepted by the judges, like the juice obtained from them. Positive descriptors for taste such as sweet and pomegranate flavor reached scores above 6 out of 9. Additionally, the visual evaluation descriptor color reached high scores for both arils and juice on the fresh fruit. All negative descriptors, such as acid, bitter, or astringent, marked scores below or equal to 2, on a scale from 1 to 9, on the fresh fruit.

**Figure 5:**
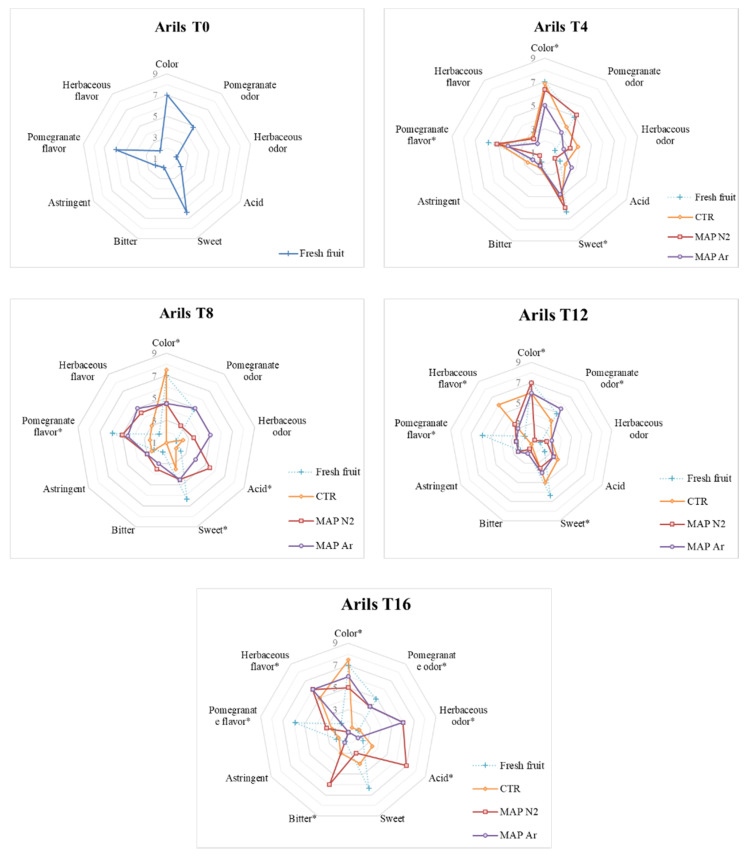
Sensory analysis performed on the arils treated with MAP (CTR: 20.9% O^2^ + 0.04% CO^2^ (passive-MAP); MAPN2: 10% O^2^ + 5% CO^2^ + 85% N2; MAPAr: 10% O^2^ + 5% CO^2^ + 85% Ar). Analyses were carried out after storage for 0 (T_0_), 4 (T_4_), 8 (T_8_), 12 (T_12_), and 16 (T_16_) days at 4 ± 1 °C and 90 ± 5% RH. Scores of the fresh fruit, marked with +, are shown at all evaluation times for reference purpose. * indicates the presence of significant differences between treatments at the same analysis time.

**Figure 6:**
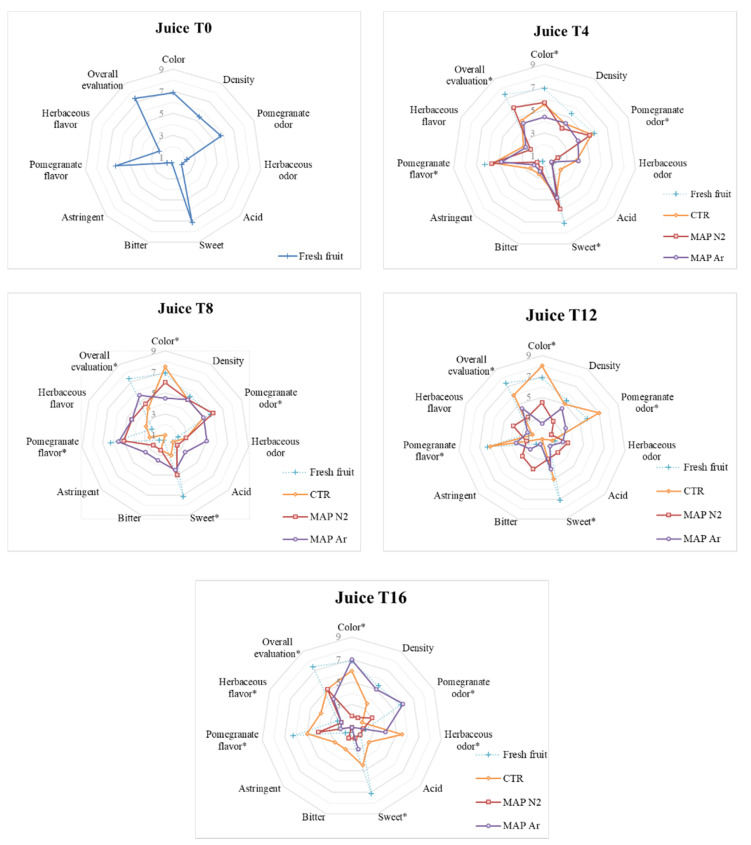
Sensory analysis performed on the juice arils treated with MAP (CTR: 20.9% O^2^ + 0.04% CO^2^ (passive-MAP); MAPN2: 10% O^2^ + 5% CO^2^ + 85% N2; MAPAr: 10% O^2^ + 5% CO^2^ + 85% Ar). Analyses were carried out after storage for 0 (T_0_), 4 (T_4_), 8 (T_8_), 12 (T_12_), and 16 (T_16_) days at 4 ± 1 °C and 90 ± 5% RH. Scores of the fresh fruit, marked with +, are shown at all evaluation times for reference purpose. * indicates the presence of significant differences between treatments at the same analysis time.

As can be observed in Figure 5 and Figure 6, at time T_4_, no major differences emerged among the treatments, but CTR and MAPN_2_ are the ones where color reaches the highest score both for arils and juice.

After eight days of storage, the negative descriptors acid and herbaceous odor reach values above 5 in MAPN2 arils, while MAPAr maintains intermediate values below 5 for all descriptors, and CTR shows the best color in both arils and juice.

Differences among treatments start to emerge at T_12_, where herbaceous flavor is registered in CTR arils, and pomegranate odor is not detected in MAPN2 arils, while in the juice of the same treatment, the negative descriptor for taste bitter is perceived by the judges. MAPAr arils, instead, maintain pomegranate odor, and the juice maintains low scores for the negative descriptors.

At the last evaluation time T_16_, arils and juice of the treatment MAPN2 obtain high scores in negative descriptors bitter and acid, while the juice of the same treatment is not appreciated, obtaining values between 1 and 2 for all descriptors. CTR arils were the ones where the color was most appreciated, while the juice showed an intense herbaceous odor. MAPAr arils and juice still do not show any major defect, with negative descriptors scoring between 1 and 2.

In general, one of the most important results emerging from the sensory analysis is the absence of noticeable defects in the MAPAr treated arils, which only reach a score of 6 out of 9 for the descriptor “herbaceous odor” and “herbaceous flavor” after 16 days of storage, while all other negative descriptors reach scores lower than 5 at all evaluation times. Argon-treated arils do not show any sign of bitterness at any time. Overall, visual appearance of the arils was satisfactory for the judges until the end of the cold storage for all treatments, as the lowest scores for the color descriptor were always above 5 out of 9. This represents an improvement compared to previous studies on MAP-treated arils, which were found to be considered visually unacceptable for consumers after 10 or 14 storage days [23,24]. The treatment where negative descriptors for the juice reached the highest scores, highlighting the appearance of sensory defects as storage proceeded, was MAPN_2_.

## 4. Conclusions

Our results demonstrated the ability of argon to maintain the quality characteristics of pomegranate arils (*Punica granatum* cv. Wonderful), and this first insight on the use of argon-based gaseous mixture for the modified atmosphere packaging preservation of pomegranate arils provided encouraging results. In particular, the treatment MAP Ar was better than a commonly used MAP N_2_ mixture in maintaining a high juice content of the arils and TSSC/TA ratio, limiting the accumulation of CO_2_ in the storing packages, and extending visual acceptability of the arils and their juice after cold storage. Furthermore, Ar treatments delay the browning process while maintaining the color characteristics of the fresh product and inhibit the growth of microorganisms for as long as two weeks under cold storage conditions.

Therefore, the use of argon can represent a promising opportunity to preserve pomegranate arils in ready-to-eat packages, so as to meet the needs of the growing number of consumers seeking “functional food”. The positive effects of Ar treatment on aril quality could be due to the presence of Ar clathrates, but further research is needed to obtain more evidence on the elucidation of the mechanism of MAP treatment with Ar.

Finally, sensory analysis supports the results of instrumental analysis and confirms that MAP treatment with Ar does not alter the taste and maintains the characteristics of both the arils and their juice until day 16 of storage. Therefore, such treatment could be suggested to food industries wanting to offer their consumers a product with excellent physico-chemical and sensory characteristics, as our analyses confirmed, and that is microbiologically safe. It is important, however, that the arils are treated in accordance with the recommended food safety management systems practices, with particular attention to the manipulation of the raw material.

## Figures and Tables

**Table 1:** Physical characteristics of the fruits of pomegranate from which the arils were extracted. Values are expressed as mean ± Standard Deviation (n = 10).

Fruit Weight (g)		420.14 ± 9.44
Fruit Length—Longitudinal Diameter (cm)		6.33 ± 0.39
Fruit Width—Transverse Diameter (cm)		8.63 ± 0.66
Fruit Volume (cm^3^)		156.67 ± 20.82
Shape Index		1.36 ± 0.40
Skin Color	L*	59.37 ± 0.97
	a*	29.87 ± 1.56
	b*	35.42 ± 4.00
Skin Weight (g)		147.66 ± 7.36
Skin Thickness (cm)		0.23 ± 0.20
Skin Index		0.35 ± 0.10

**Table 2:** Characteristics of the pomegranate arils prior to treatment and storage. Values are expressed as mean ± standard deviation (n = 10).

Number of Arils		644.67 ± 32.20
Weight of Arils per fruit (g)		264.47 ± 7.77
Weight of 20 Arils (g)		6.41 ± 2.23
Aril Height—Longitudinal Diameter (cm)		1.14 ± 0.07
Aril Width—Transverse Diameter (cm)		0.68 ± 0.13
Aril Yield (Fruit Weight—Skin Weight) (g)		272.48 ± 15.28
Aril Color	L*	28.78 ± 6.78
	a*	15.88 ± 2.62
	b*	28.78 ± 3.70
Weight of 20 Seeds (g)		1.09 ± 0.08

**Table 3:** Physico-chemical characteristics of the juice extracted from the arils prior to treatment and storage. Values are expressed as mean ± standard deviation (n = 10).

Juice Content (mL/100 g)	148 ± 4.58
Total Soluble Solids Content (°Brix)	15.4 ± 0.53
Titratable Acidity (g L^−1^ citric acid)	0.14 ± 0.04
pH	4.17 ± 0.06

**Table 4:** Evolution of the CIE L*a*b* coordinates measured on the arils and their values of color difference, chroma, and hue angle (lightness (L*); redness (a*); yellowness (b*); color difference (ΔE); chroma (C*); hue angle (*h*°, rad)) treated with modified atmosphere packaging (CTR: 20.9% O_2_ + 0.04% CO_2_ (passive-MAP); MAPN_2_: 10% O_2_ + 5% CO_2_ + 85% N_2_; MAPAr: 10% O_2_ + 5% CO_2_ + 85% Ar). Different letters indicate statistical differences (*p* ≤ 0.05) between treatments for the same storage times. Letter “a” denotes the highest value, “ns” denotes no significant difference. Values were recorded at 0 (T_0_), 4 (T4), 8 (T_8_), 12 (T_12_), and 16 (T_16_) days at 4 ± 1 °C and 90 ± 5% RH. Data correspond to the means ± SD.

Storage Days		L*	a*	b*	ΔE	C*	*h*° (rad)
0	Fresh fruit	28.78	15.88	10.60	-	19.09	0.59
4	CTR	17.57 ± 0.84 ^c^	16.67 ± 1.75 ^b^	9.58 ± 1.34 ^ns^	11.28 ^a^	19.23 ^b^	0.52 ^b^
	MAP N2	22.69 ± 8.31 ^b^	15.14 ± 2.18 ^b^	11.48 ± 2.16 ^ns^	6.20 ^b^	19.00 ^b^	0.65 ^a^
	MAP Ar	30.08 ± 5.05 ^a^	18.75 ± 2.79 ^a^	10.98 ± 1.77 ^ns^	3.17 ^c^	21.73 ^a^	0.53 ^b^
8	CTR	24.13 ± 4.82 ^c^	19.39 ± 0.84 ^a^	10.03 ± 2.10 ^b^	5.85 ^b^	21.83 ^b^	0.48 ^b^
	MAP N2	36.20 ± 11.68 ^a^	20.93 ± 0.84 ^a^	13.66 ± 5.63 ^a^	9.48 ^a^	24.99 ^a^	0.58 ^a^
	MAP Ar	28.42 ± 8.61 ^b^	17.22 ± 0.88 ^b^	11.53 ± 1.86 ^b^	1.67 ^c^	20.72 ^b^	0.59 ^a^
12	CTR	25.58 ± 2.66 ^a^	19.05 ± 1.93 ^a^	10.23 ± 3.10 ^a^	4.52 ^a^	21.62 ^a^	0.49 ^c^
	MAP N2	23.71 ± 0.57 ^b^	14.12 ± 1.64 ^b^	8.9 ± 1.30 ^b^	5.62 ^a^	16.71 ^b^	0.56 ^b^
	MAP Ar	26.82 ± 7.11 ^a^	16.68 ± 6.62 ^b^	11.80 ± 2.73 ^a^	2.43 ^b^	20.43 ^a^	0.62 ^a^
16	CTR	18.58 ± 1.43 ^b^	16.59 ± 3.15 ^ns^	10.29 ± 1.43 ^ns^	10.23 ^a^	19.52 ^ns^	0.56 ^b^
	MAP N2	24.81 ± 4.36 ^a^	15.79 ± 1.29 ^ns^	10.50 ± 1.02 ^ns^	3.97 ^b^	18.96 ^ns^	0.59 ^a^
	MAP Ar	20.74 ± 2.30 ^b^	17.15 ± 1.77 ^ns^	10.11 ± 2.00 ^ns^	8.15 ^a^	19.91 ^ns^	0.53 ^b^

**Table 5:** Total mesophilic (TMM) and psychrotrophic bacterial (TPM) loads of the fruit samples treated with MAP (CTR: 20.9% O_2_ + 0.04% CO_2_ (passive-MAP); MAPN_2_: 10% O_2_ + 5% CO_2_ + 85% N_2_; MAPAr: 10% O_2_ + 5% CO_2_ + 85% Ar). Units are log CFU/g. Results indicate mean values ± SD. Different letters indicate statistical differences (*p* ≤ 0.05) between treatments for the same storage times. Letter “a” denotes the highest value, “ns” denotes no significant difference. Values were recorded at 0 (T_0_), 4 (T_4_), 8 (T_8_), 12 (T_12_), and 16 (T_16_) days at 4 ± 1 °C and 90 ± 5% RH.

Treatments	TMM					TPM				
	T_0_	T_4_	T_8_	T_12_	T_16_	T_0_	T_4_	T_8_	T_12_	T_16_
CTR	<2 ^ns^	<2 ^ns^	<2 ^ns^	3.10 ± 0.10 ^a^	4.00 ± 0.11 ^a^	<2 ^ns^	<2 ^ns^	<2 ^ns^	<2 ^ns^	4.40 ± 0.09 ^a^
MAPN_2_	<2 ^ns^	<2 ^ns^	<2 ^ns^	2.67 ± 0.19 ^b^	3.65 ± 0.20 ^b^	<2 ^ns^	<2 ^ns^	<2 ^ns^	<2 ^ns^	3.90 ± 0.08 ^b^
MAPAr	<2 ^ns^	<2 ^ns^	<2 ^ns^	2.37 ± 0.20 ^b^	3.25 ± 0.17 ^c^	<2 ^ns^	<2 ^ns^	<2 ^ns^	<2 ^ns^	3.70 ± 0.10 ^c^

**Table 6:** Yeast and mold loads of fruit samples treated with MAP (CTR: 20.9% O_2_ + 0.04% CO_2_ (passive-MAP); MAPN_2_: 10% O_2_ + 5% CO_2_ + 85% N_2_; MAPAr: 10% O_2_ + 5% CO_2_ + 85% Ar). Units are log CFU/g. Results indicate mean values ± SD. Different letters indicate statistical differences (*p* ≤ 0.05) between treatments for the same storage times. Letter “a” denotes the highest value, “ns” denotes no significant difference. Values were recorded at 0 (T_0_), 4 (T_4_), 8 (T_8_), 12 (T_12_), and 16 (T_16_) days at 4 ± 1 °C and 90 ± 5% RH.

Treatments	Yeast					Molds				
	T_0_	T_4_	T_8_	T_12_	T_16_	T_0_	T_4_	T_8_	T_12_	T_16_
CTR	<2 ^ns^	<2 ^ns^	<2 ^ns^	3.10 ± 0.10 ^a^	3.70 ± 0.11	<2 ^a^	<2 ^a^	<2 ^a^	<2 ^a^	3.00 ± 0.09 ^a^
MAPN_2_	<2 ^ns^	<2 ^ns^	<2 ^ns^	2.20 ± 0.19 ^b^	2.40 ± 0.20	<2 ^a^	<2 ^a^	<2 ^a^	<2 ^a^	2.80 ± 0.08 ^b^
MAPAr	<2 ^ns^	<2 ^ns^	<2 ^ns^	2.00± 0.20 ^b^	2.20 ± 0.17	<2 ^a^	<2 ^a^	<2 ^a^	<2 ^a^	2.60 ± 0.10 ^c^

## Data Availability

The data presented in this study are available on request from the corresponding author. The data are not publicly available due to privacy policy of the Authors’ Institution.
